# Bioinspired Oxidation-Resistant Catechol-like Sliding Ring Polyrotaxane Hydrogels

**DOI:** 10.3390/gels9020085

**Published:** 2023-01-19

**Authors:** M. Isabel Rial-Hermida, Dora C. S. Costa, Lan Jiang, João M. M. Rodrigues, Kohzo Ito, João F. Mano

**Affiliations:** 1 CICECO—Aveiro Institute of Materials, Chemistry Department, University of Aveiro, 3810-193 Aveiro, Portugal; 2 Department of Advanced Materials Science, Graduate School of Frontier Sciences, The University of Tokyo, Kashiwa-shi 277-8561, Chiba, Japan

**Keywords:** oxidation-resistant, polyrotaxanes, non-covalent bounding, hydroxypyridinone, sliding-ring hydrogels

## Abstract

Adaptable hydrogels have been used in the biomedical field to address several pathologies, especially those regarding tissue defects. Here, we describe unprecedented catechol-like functionalized polyrotaxane (PR) polymers able to form hydrogels. PR were functionalized with the incorporation of hydroxypyridinone (HOPO) moieties into the polymer backbone, with a degree of substitution from 4 to 22%, depending on the PR type. The hydrogels form through the functionalized supramolecular systems when in contact with a Fe(III) solution. Despite the hydrogel formation being at physiological pH (7.4), the HOPO derivatives are extremely resistant to oxidation, unlike common catechols; consequently, they prevent the formation of quinones, which can lead to irreversible bounds within the matrix. The resulting hydrogels demonstrated properties lead to unique hydrogels with improved mechanical behavior obtained by metallic coordination crosslinking, due to the synergies of the sliding-ring PR and the non-covalent (reversible) catechol analogues. Following this strategy, we successfully developed innovative, cytocompatible, oxidative-resistant, and reversible crosslinked hydrogels, with the potential of being used as structural self-materials for a variety of applications, including in the biomedical field.

## 1. Introduction

Several works have explored the possibility of mimicking the byssus threads of mussels, such as *Mytilus edulis*. Studies have found that this type of mollusk shows strong adhesion ability to rocks in sea conditions, that is, in wet environments and with external force against the attachment. This phenomenon is possibly due to the adhesive properties present in the mussel’s secreted protein that contains the 3,4-dihydroxy-L-phenylalanine (DOPA) amino acid precursor in its structure, which is the key factor in the adhesion capacity of mussels [1]. Due to the high versatility, good biocompatibility, and excellent adhesion performance, mussel-inspired catechol strategies have been widely explored through chemical modification of several molecules, such as synthetic and natural polymers, to improve wet adhesion, adherence to surfaces, and self-healing capacities [2,3,4,5,6,7]. However, the easy and fast oxidation of these entities requires the use of acidic conditions for the manipulation of these compounds, which are not optimal when biomedical applications are intended [8].

Regarding this field, a plethora of polymeric-based systems have been developed due to their similarity to the extracellular matrix, such as nanofibers [9] or several class of composite hydrogels, combining the features of two or more polymeric systems to address the typical issues experienced with polysaccharides [10]. In particular, polymeric hydrogels have been widely proposed as artificial matrices to support new tissue development, for the encapsulation of cells, or to control the delivery of active substances [11]. Along these lines, adaptable hydrogels could be extraordinary systems to meet the needs of this field, especially in tissue engineering [12]. Adaptable hydrogels are based on reversible crosslinks. These reversible interactions are important for injectable systems due to the breaking and re-establishment of the linkages, allowing the adaptable hydrogels to be locally modified [12,13,14]. In this sense, metallic coordination is an excellent strategy to reach this specific aim, enabling to control of different properties such as the stiffness of the hydrogel [15,16]. This type of supramolecular bonding can be modulated at specific pHs, which confers the biomaterial with the capacity to be pH-sensitive and widely versatile [13,17]. In these cases, the oxidation of molecules plays a very important role in the stability of the coordination complexes, which directly influences the formation of the system. Usually, this type of hydrogels demonstrates fragile or poor mechanical properties when this coordination is implemented alone; however, it has an extraordinary potential to control the stiffness and the ability to make reversible systems [16,18]. For that, coordination crosslinks are commonly employed, forming part of double networking (DN) hydrogels, owing to their capacity for improving the toughness of polymeric systems [2,19,20,21].

Additionally, hydrogels based on sliding-ring polyrotaxanes (PR) have been demonstrated as having very distinct characteristics when subjected to a loading force: high toughness and evenly distributed stress improve the mechanical properties. This phenomenon occurs because of the novel topological design, in which the cyclodextrins (CDs) inserted along the polyethylene glycol (PEG) chain have the capacity to slide over the polymer chain. By expanding or limiting the range of the CDs rings’ sliding motion (for example, by controlling the length of the polymer backbone or the number of CDs in each PEG chain), the mechanical properties of the PRs, such as flexibility or elasticity, can be tuned [22]. For those reasons, they have been explored in several fields, including tissue engineering and drug delivery [23]. Additionally, these supramolecular entities can form hydrogels with mechanical properties similar to those of cartilage, due to their capacity to diffuse the applied forces [23,24].

The possibility of dynamic interchain crosslinking creates the potential for tuning these polymers for numerous applications, modifying their mechanical properties by the type of bond that is applied in substituent groups present in the polymer backbone [23,25]. In this regard, several studies have been conducted on PR networks, such as PR with self-healing behavior by host–guest interactions [26,27].

For the potential development of innovative adhesives and to overcome the easy and fast oxidation issues of the common catechols, different hydroxypyridinones (HOPOs) have been explored [28,29]. These molecules are oxidation-resistant catechol analogues and have been demonstrated as more resistant to oxidation than DOPA [28,29]. Moreover, they are stable and biocompatible and able to promote the formation of robust, but, at the same time, they are reversible, polymeric networks [28].

**Figure 1:**
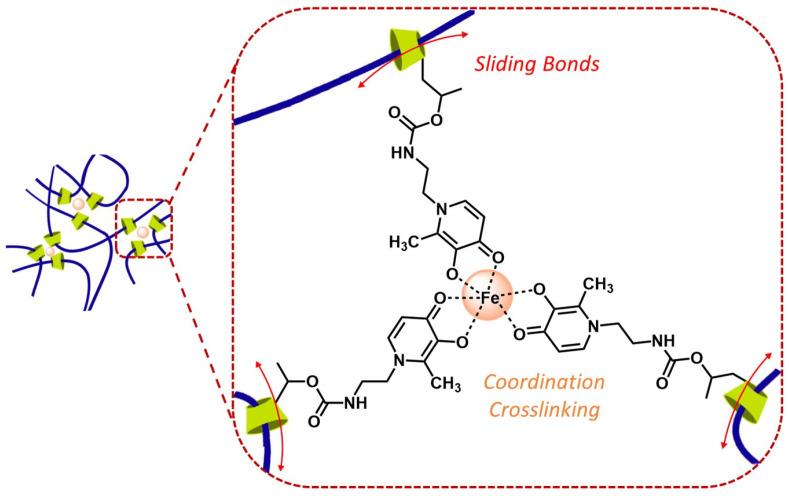
Schematic illustration of modified sliding-ring PR-HOPO networks.

On the way to exploring novel biocompatible hydrogels with well-distributed stress forces, we report, in a straightforward approach, a system combining the unique features of sliding-ring PRs with the special properties of HOPOs (Figure 1). Thus, we developed a library of biomimetic, robust, flexible, self-healing, and oxidation-resistant hydrogels, based on functionalized PR with HOPO moieties, solving the common issues of the easy and rapid oxidation in this type of system.

## 2. Results and Discussion

### 2.1. Preparation and Characterization of Novel Polyrotaxane-Hydroxypyridinones (PR-HOPO) Network

**Figure 2:**
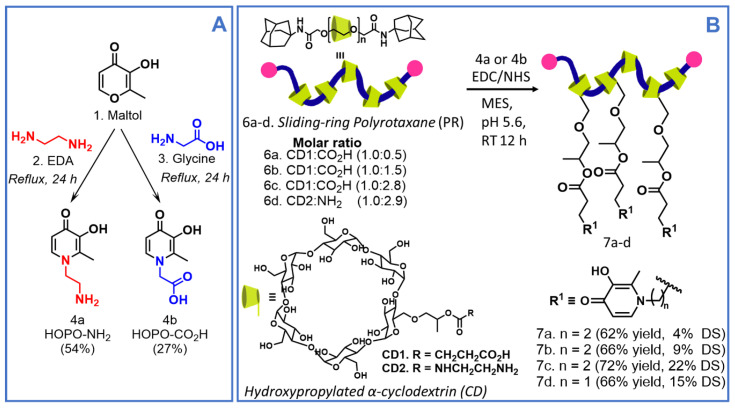
Synthetic pathway for the preparation of (**A**) HOPOs and (**B**) polyrotaxane-hydroxypyridinone (PR-HOPO) polymers (**7a**–**d**).

The novel PR-based materials functionalized with oxidation-resistant catechol-like moieties were prepared in two reaction steps, following the synthetic pathway outlined in Figure 2. Firstly, based on previous procedures [29,30], we prepared two different HOPOs precursors: (a) HOPO with a terminal amino group (**4a**, HOPO-NH_2_) [29] and (b) HOPO with a terminal carboxylic group (**4b**, HOPO-CO_2_H) [30].

Briefly, HOPO-NH_2_ (**4a**) was prepared from maltol (1) through a ring-opening/closing reaction with ethylenediamine (2. EDA), under alkaline conditions. After 24 h under reflux, **4a** was obtained by filtration, as light brown solid in 54% yield. Similarly, HOPO-CO_2_H (**4b**) was synthesized from maltol (1) and glycine (3). After 24 h under reflux, **4b** was obtained as a beige solid with a 27% yield (Figure 2A). In order to confirm their structures, ^1^H (Figures S1 and S3) and ^13^C NMR (Figures S2–S4) spectra analyses were carried out, and the obtained spectra were in good agreement with already reported data [29,30].

Previously, as reported by Ito et al. [31,32], PRs containing terminal carboxylic groups with three different molar ratios CD:CO2H (**6a**–**c**, Figure 2B) and with terminal amino groups (**6d**, Figure 2B), were functionalized with the prepared HOPOs (**4a** or **4b**) to avoid quick oxidation, an issue widely reported for traditional catechol moieties [14,30]. To do so, PR **6a**–**c** and **6d** were coupled with HOPOs **4a** and **4b**, respectively, by EDC/NHS coupling reaction in MES buffer at pH 5.6. After a 12 h reaction period at room temperature (RT), the products were dialyzed and freeze-dried. All PR-HOPO (**7a**–**d**) polymers were obtained as white/slightly yellow solids, with a substitution degree (DS) of 4%, 9%, 22%, and 15% (*w*/*w*), respectively, confirmed by ^1^H NMR and quantified by UV–Vis spectroscopy (see 1.2, ESI) [29,30]. Comparing the ^1^H NMR spectra of the respective HOPO precursor (**4a** or **4b**), PRs **6a**–**d**, and functionalized PRs with HOPO **7a**–**d**, it was possible to identify two new doublets around 6.52 ppm (*J* = 7.1 Hz, i.e., for **7c**) and 7.59 ppm (*J* = 7.1 Hz, i.e., for **7c**), the characteristic signals corresponding to protons H-5 and H-6 from the HOPO precursor, respectively, which confirmed the HOPO moieties’ insertion into the PRs chain (Figures S5–S8).

### 2.2. Preparation and Characterization of PR-HOPO Hydrogels

**Figure 3:**
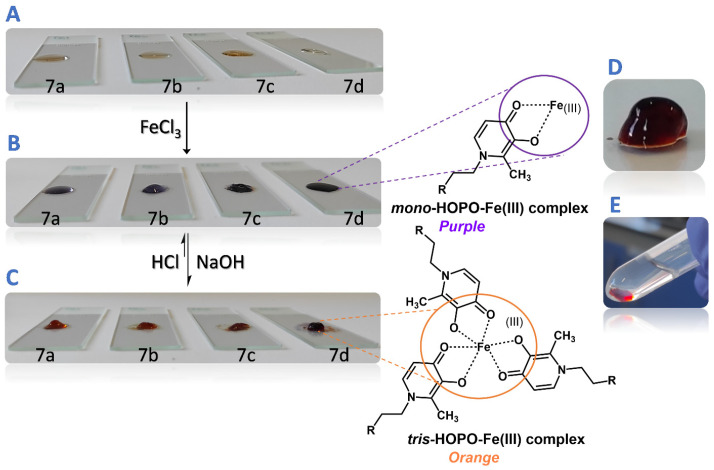
Reversible formation of PR-HOPO-based hydrogels; (**A**) 10% (*w*/*v*) **7a**–**d** suspensions in MES buffer at pH 5.6 were deposited above a glass slide; (**B**) after the addition of 20 mM FeCl_3_ solution, suspensions changed to purple, demonstrating *mono*-coordination had taken place; (**C**) pH of suspensions was increased until physiological pH was achieved, and the suspensions turned orange due to the trivalent coordination; (**D**) and (**E**) hydrogels had stable networks after 24 h in PBS pH 7.4 solution.

To evaluate the hydrogel formation (Figure 3), polymers **7a**–**d** were dispersed in MES buffer at pH 5.6 and 10% (*w*/*v*), and were prepared following the concentrations proposed by Holten-Andersen et al. [15], with slight modifications. Final hydrogels were composed by 1/2 of the volume of the dispersion of **7a**–**d**, 1/3 of the volume of the metal solution (20 mM FeCl_3_), and 1/6 of the volume of NaOH (0.1 M), in order to increase the pH of the medium to *ca.* 7.4 to promote the tris-catecholate complex between the metal and HOPO moieties [33]. The hydrogel formation was observed almost immediately after pH changing (Video S1). A semisolids pH meter was used to monitor the pH throughout the whole process, avoiding a pH greater than 7.4. All developed systems were demonstrated to be robust and stable in PBS solution at pH 7.4 for 24 h. The hydrogels showed soft adhesion properties and an interesting capacity to self-heal after a scratch (Video S2).

**Figure 4:**
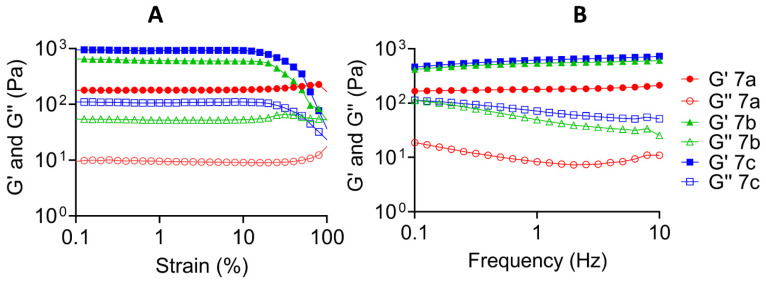
Rheological properties of the different formulations based on PR-HOPO **7a**–**c**. (**A**) Strain sweep measurements at 1 Hz (fixed), and (**B**) frequency sweep measurements at 1% of strain (fixed). Hydrogel networks showed stability in all formulations.

The PR-HOPO derivatives **7a**–**c** led to hydrogels with elastic modulus (1 kPa) greater than the already reported ones modified with traditional catechol moieties [17]. In all cases, the storage modulus was greater than the loss modulus, which is a classic behavior of gel-like materials [34], and no changes in the moduli appeared after the cut (Figure 4 and Figure S9). This behavior confirmed the hypothesis of reversible hydrogels formation, just by metallic coordination.

Moreover, a clear difference between the four formulations was observed, according to the substitution degree of each PR. Another important parameter that should be taken into account is the number of CDs along the PEG chain [35], because this is directly related to the flexibility and elasticity of the system. In this study, as a proof of concept, all PRs contained 100 CDs for each PEG chain (Mw 35,000 Da). As mentioned above, the PRs were functionalized with amino (**6d**) and carboxylic groups in several degrees (**6a** was less substituted and **6c** was more substituted; Figure 2B). Then, the obtained insertion of HOPO groups was performed according to the initial available amount of amino or carboxylic groups of **6a**–**d**, i.e., **7a** had the smallest DS and **7c** the higher one. Consequently, PR-HOPO **7b** and **7c** demonstrated a greater elastic modulus than PR-HOPO **7a** (Figure S10). Additionally, by the DS of HOPO groups in the PRs chain, it was possible to determine the number of possible metallic coordinations that these supramolecular systems can form. Thus, we could produce mechanically versatile hydrogels that are only dependent on the availability of groups to take part in the metallic coordination. This is especially important and opens a way to new approaches for the development of structural hydrogels, for example, for engineering substitute tissues and potential tissue substitutes, retaining the properties of the native ones.

### 2.3. Assessment of Cell Viability upon Contact with PR-HOPO Formulations

**Figure 5:**
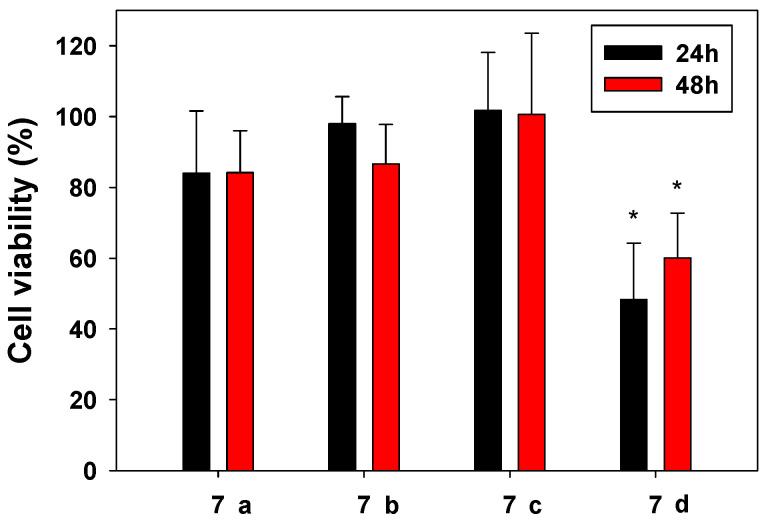
Cell viability of osteoblast cells when in contact with 100% extracts from the different prepared hydrogels (**7a**–**d**) compared with controls, after 24 and 48 h at 37 °C, 95% RH, and 5% CO_2_. Tests were carried out in triplicate. * Statistical differences were found in formulation **7d** when compared with the rest of the formulations.

The cytocompatibility of the hydrogels was assessed with SAOS-2 cells (Figure 5). The extracts from the samples were performed under the guidelines of ISO standard 10993:5 (cytotoxicity of medical devices). After 24 and 48 h, a cell proliferation test (MTS) was carried out following the instructions of the manufacturer. Hydrogels based on **7a**, **7b**, and **7c** showed a 70% proliferation when compared with controls in all formulations, classified as non-cytotoxic according to ISO 10993:5. Statistical analysis demonstrated that formulation **7d** differed from the other samples, with lower values of cell proliferation (confidence level of 95%, significance level (***α***) of 0.05). On the other hand, there were no statistical differences in the viability of the cells for the same sample at 24 and 48 h. Commonly, for the crosslinking of these types of network, the pH must be increased to around 8–9 [15]. Due to the needs of this pH, we could hypothesize the non-compatibility of the systems. In our case, we kept complete control by checking the pH from the synthesis of the PR-HOPO until the metallic coordination, maintaining it at 5.6 during the reaction process to 7.4 to promote the metallic coordination. 

## 3. Conclusions

Overall, biocompatible, cutting-edge, oxidation-resistant, and self-healing hydrogels were developed, taking advantage of functionalized sliding-ring PRs with HOPO moieties and avoiding the common oxidation issues. This completely novel macromolecular design results in hydrogels with the capacity to distribute the typical stresses among the polymer chains subject to compression stress, along the entire volume of the hydrogel through reversible free movements of CDs. It is important to note that the developed hydrogels were obtained with just one network; yet, they showed interesting mechanical properties. The possibility to modulate the crosslinking degree and tune the properties of the hydrogels makes this approach a versatile system. Here, we proved that PR-HOPO formulations are able to create reversible systems just by coordination crosslinking. The capacity to maintain the polymeric network integrity when the coordination of the HOPO is achieved at physiological pH makes them compatible with osteoblast cells. These features give an added value to these systems, markedly differentiated from the existing systems based on the same chemistry.

## 4. Materials and Methods

### 4.1. Materials

Ethylenediamine (EDA), maltol, *N*-(3-dimethylaminopropyl)-*N*′-ethylcarbodiimide hydrochloride (EDC) [≥98.0% (AT)], *N*-hydroxysuccinimide (NHS), 2-(*N*-morpholino)ethanesulfonic acid buffer (MES), and phosphate-buffered saline (PBS) were purchased from Sigma Aldrich (Taufkirchen, Germany) and used without any further purification. Methanol (p.a., 99.9%) was acquired from Fisher Scientific (Waltham, MA, USA). Deuterium oxide (99.8%) was bought from TCI Chemicals (Zwijndrecht, Belgium). Sodium deuteroxide (40 wt.% solution in D_2_O, 99+%) was obtained from Acros (Thermo Fisher Scientific, Waltham, MA, USA). Spectra/Por dialysis membranes with a molecular weight cut-off (MWCO) of 3.5 kDa were supplied from Spectrum Laboratories (Waterford, Ireland). MTS cell proliferation tests (Kit ab197010) were acquired from Abcam (Cambridge, UK). ^1^H and ^13^C NMR spectra were recorded using a Bruker Avance-300 spectrometer (Bruker Daltonics, Billerica, MA, USA) at 300.13 MHz and 75.47 MHz, respectively, using tetramethylsilane (TMS) as an internal reference. The chemical shifts are expressed in δ (ppm) and the coupling constants (*J*) in Hz. Melting point (m.p.) was measured with a Büchi Melting point B-540 apparatus (Buchi Laboratory Equipment, Flawil, Switzerland), and values were uncorrected. The freeze-dried products were obtained on a LyoQuest Plus Freeze-dryer (Telstar, Terrasa, Spain). The pH of the hydrogels was monitored with a pH meter with a penetration probe for semisolid systems (Dominique Dutscher, Bernolsheim, France). The UV–Vis spectra and MTS assays were recorded on a Synergy HTX Microplate Reader (Agilent Technologies, Santa Clara, CA, USA), using a 96-well quartz microplate. Rheological tests were performed with a Kinexus Lab+ rheometer (Malvern Panalytical, Malvern, UK), by using a 20 mm diameter parallel plate geometry and a 0.5 mm gap at 25 °C. To determine the linear viscoelastic region (LVR), strain amplitude sweep measurements (0.1 to 1000%) were performed at a frequency of 1.0 Hz. Oscillatory frequency sweep measurements (0.01 to 100 Hz) were then conducted at constant 1% strain amplitude to determine the storage (G′) and loss (G″) moduli.

### 4.2. Synthesis and Characterization of Novel Polyrotaxane-Hydroxypyridinones (PR-HOPO) 

#### 4.2.1. General Procedure for the Preparation of HOPO **4a** and **4b**

***1-(2-aminoethyl)-3-hydroxy-2-methylpyridin-4(1H)-one (4a)***. Compound **4a** was synthesized as follows, based on a previously reported method [29,30]. Maltol (1) (5.0 g, 39.6 mmol, 1.0 eqv) was added to hot water (100 mL) in the presence of EDA (2) (4.0 mL, 59.4 mmol, 1.5 eqv). To promote total dissolution, the mixture was stirred and heated to 80 °C. The pH solution was adjusted to 10 by dropwise addition of 6 M HCl solution and monitored by pH paper. The mixture was brought to reflux and stirred for 24 h and then cooled to RT. All water was removed under reduced pressure. The crude product was dissolved in 20 mL of distilled water, and its pH was adjusted to ~7 with the addition of 6 M HCl solution. All water was again evaporated. The product was precipitated by the addition of methanol/diethyl ether. A light brown precipitate was isolated by filtration, washed with cold methanol, and dried in an oven for 12 h, at 60 °C, to yield compound **4a** (3.6 g, 54%). m.p.: decomposed >275 °C. ^1^H NMR (300.0 MHz, D_2_O) δ (ppm): 2.42 (s, 3H, H-7), 3.41 (t, *J* = 6.8 Hz, 2H, H-3′), 4.41 (t, *J* = 6.8 Hz, 2H, H-2′), 6.51 (d, *J* = 7.3 Hz, 1H, H-5), 7.65 (d, *J* = 7.3 Hz, 1H, H-6). ^13^C NMR (75.0 MHz, D_2_O) δ (ppm): 11.5 (C-7), 38.6 (C-2′), 50.8 (C-3′), 112.9 (C-5), 134.3 (C-2), 138.6 (C-6), 145.0 (C-3), 169.5 (C-4). 

***2-(3-hydroxy-2-methyl-4-oxopyridin-1-(4H)-yl)acetic acid (4b).*** The procedure for the synthesis of compound 4b was adapted from a procedure reported in the literature [29,30]: Maltol (1) (7.5 g, 59.5 mmol, 1.0 eqv) was added to hot water (150 mL) in the presence of glycine (3) (8.9 g, 119.0 mmol, 2.0 eqv). To promote total dissolution, the mixture was stirred and heated to 80 °C. The pH of the solution was increased to 9 by the dropwise addition of 6 M NaOH solution and monitored by pH paper. The mixture was brought to reflux and maintained for 24 h. The reaction mixture was cooled to room temperature (RT), and almost all water was removed under reduced pressure using a rotary evaporator. The pH of the crude product was brought to ~3 with the addition of a 6 M HCl solution, at which time a beige solid precipitated was formed. The precipitate was isolated by filtration and was subsequently recrystallized from hot water and stored at 4 °C overnight. The resulting solid was filtered off, washed with distilled water, and dried in an oven at 60 °C for 12 h, to yield a beige solid, which was identified as compound **4b** (3.5 g, 27%). m.p.: 256.5–257.4 °C. ^1^H NMR (300.0 MHz, D_2_O/NaOD) δ (ppm): 2.25 (s, 3H, H-7), 4.59 (s, 2H, H-2′), 6.41 (d, *J* = 7.0 Hz, 1H, H-6), 7.34 (d, *J* = 7.0 Hz, 1H, H-5). ^13^C NMR (75.0 MHz, D_2_O/NaOD) δ (ppm): 11.9 (C-7), 58.2 (C-2′), 111.7 (C-5), 135.4 (C-2), 135.6 (C-6), 151.3 (C-3), 171.5 (C-4), 174.6 (C-1′).

#### 4.2.2. General Procedure for Polyrotaxane-Hydroxypyridinones (PR-HOPO) Functionalization

HOPO-NH_2_ (**4a**) and HOPO-CO_2_H (**4b**) were conjugated onto the PR-CO_2_H (**6a**–**c**) and PR-NH_2_ (**6d**) chains, respectively, by EDC/NHS coupling reactions. Thus, PR-CO_2_H or PR-NH_2_ (100 mg, 1 eqv) was dissolved in 50 mL of MES buffer at pH 5.6 into a reaction vial. Then, EDC (287.51 mg, 6.0 eqv) was dissolved in 10 mL of MES buffer at pH 5.6 and added to the previous reaction vial for 10 min. Likewise, NHS (172.63 mg, 6.0 eqv) was dissolved in 10 mL of MES buffer pH 5.6 and added to the reaction mixture. After 15 min, HOPO-NH_2_ (**4a**) or HOPO-CO2H (**4b**) (50.74 mg or 55.85 mg respectively, 6.0 eqv), previously dissolved in 10 mL of MES buffer pH 5.6, was added to the previous reaction mixture and left to react at RT for 12 h. Then, the resulting reaction solution was transferred to dialysis bags and was dialyzed against MES buffer pH 5.6 for 5 days under stirring at RT. The MES buffer was changed twice per day. PR-HOPO derivatives (**7a**–**d**) were obtained by freeze-drying as a pale white/slightly yellow solid and stored at −20 °C. 

**(7a)** Pale white/slightly yellow solid (93 mg, 62% yield and DS = 4% *w*/*w*). ^1^H NMR (300.0 MHz, D_2_O) δ (ppm): 1.15 (s), 2.45–2.69 (m), 2.88 (bs), 3.33–3.38 (m), 3.52–3.57 (m), 3.69 (bs), 3.97 (bs), 5.08 (bs), 5.21 (bs), 6.54 (bs), 7.59 (d, *J* = 5.6 Hz).

**(7b)** Pale white/slightly yellow solid (99 mg, 66% yield and DS = 9% *w*/*w*). ^1^H NMR (300.0 MHz, D_2_O) δ (ppm): 1.15 (s), 2.45–2.69 (m), 2.88 (bs), 3.33–3.38 (m), 3.52–3.57 (m), 3.69 (bs), 3.97 (bs), 5.08 (bs), 5.22 (bs), 6.75 (bs), 7.72 (bs).

**(7c)** Pale white/slightly yellow solid (108 mg, 72% yield and DS = 22% *w*/*w*). ^1^H NMR (300.0 MHz, D_2_O) δ (ppm): 1.21 (s), 2.32 (bs), 2.43 (bs), 2.59–2.73 (m), 3.18 (bs), 3.22–3.43 (m), 3.69 (s), 3.81–4.02 (m), 4.65 (s), 6.52 (d, *J* = 7.1 Hz), 7.59 (d, *J* = 7.1 Hz), 8.08 (bs).

**(7d)** Pale white/slightly yellow solid (102 mg, 65% yield and DS = 15% *w*/*w*). ^1^H NMR (300.0 MHz, D_2_O) δ (ppm): 1.21 (bs), 2.31 (s), 2.44 (bs), 2.65 (bs), 3.23 (bs), 3.27–3.45 (m), 3.68 (s), 3.91 (bs), 4.64 (bs), 6.51 (d, *J* = 6.1 Hz), 7.59 (bs), 8.07 (bs).

#### 4.2.3. Determination of the Degree of Substitution (DS)

The different degrees of substitution of HOPOs in the PRs were determined by UV–Vis spectroscopy at 276 nm (standard curve from 1 to 20 µg·mL^−1^ of HOPO-NH_2_ (**4a**) in ultrapure water: y = 0.05343x − 0.0062, R^2^ = 0.9991; standard curve of HOPO-CO_2_H (**4b**) in ultrapure water: y = 0.0551x − 0.0061, R^2^ = 0.9930). Solutions of 20 µg·mL^−1^ in ultrapure water of **7a**–**d** were prepared, and the absorbance at 276 nm wavelength was compared with the standard curves [29,34]. Solutions of each PR at the same concentration (20 µg·mL^−1^) were used as blanks. All measurements were conducted in triplicate.

### 4.3. Preparation and Characterization of HOPO-PR Hydrogels

Polymers **7a**–**d** were dispersed in MES buffer at pH 5.6 and 10% (*w*/*v*) to prepare the hydrogels, following the concentrations proposed by Holten-Andersen et al. [16]. The final hydrogels were composed by 1/2 of the volume of the dispersion of **7a**–**d**, 1/3 of the volume of the metal solution (20 mM FeCl_3_), and 1/6 of the volume of NaOH (0.1 M), in order to increase the pH medium at 7.4 ca. to promote the *tris*-catecholate complex between the metal and HOPO moieties [36]. The pH of the hydrogels was monitored during the formation with a pH meter with a penetration probe for semisolid systems (Dutscher). 

The elastic and the viscous moduli and the self-healing ability of prepared hydrogels were evaluated. The mechanical properties of the PR-HOPO-based hydrogels before and after a scratch were established by performing frequency and strain sweep tests. Rheological tests were performed with a Kinexus Lab+ rheometer (Malvern Panalytical), by using a 20 mm diameter parallel plate geometry and 0.5 mm gap at 25 °C. To determine the linear viscoelastic region (LVR), strain amplitude sweep measurements (0.1 to 100%) were performed at a frequency of 1.0 Hz. Oscillatory frequency sweep measurements (0.01 to 100 Hz) were then conducted at constant 1% strain amplitude to determine the storage (G′) and loss (G″) moduli.

### 4.4. Assessment of Cell Viability upon Contact with PR-HOPO Formulations

In order to establish the cytocompatibility of the PR-HOPO formulations, cell proliferation MTS tests were carried out after contact with the extracts of PR-HOPO with osteoblast SAOS-2 cells. Firstly, extracts from the samples were obtained as described in ISO standard 10993:5 and 10993:12 guidelines. A total of 500 mg of each sample was extracted in triplicate at 37 °C during 24 h in DMEM culture (1 mL). Then, 100 µL of SAOS-2 osteoblast cells suspension (concentration 200,000 cells mL^−1^) was seeded in 96-well plates. After 24 h of incubation at 37 °C, 95% relative humidity (RH), and 5% CO_2_, several concentrations (50%, 25%, 12.5%, and 6.25% *v*/*v*) extracted from the samples were added and placed in contact with the cells for 24 and 48 h. Then, a cell proliferation test (MTS Proliferation Assay Kit ab197010, Abcam) was carried out following the instructions of the manufacturer. As controls, cells seeded with complete culture medium only were used. Absorbance was measured at 490 nm on a Synergy HTX Microplate Reader using a 96-well quartz microplate. All experiments were carried out in triplicate.

### 4.5. Statistical Analysis

Results are shown as mean values and standard deviations of at least three replicates. Regression analysis for the determination of the degree of substitution was carried out using Microsoft Excel Office 365 software (Microsoft Corporation, Redmond, WA, USA). Differences in the types of PR-HOPO formulations regarding the cell proliferation were determined by applying a two-way ANOVA test (confidence level of 95%, significance level (***α***) of 0.05) and Tukey’s multiple comparison test with GraphPad Prism 8.3.0 (GraphPad Software Inc, New York, NY, USA). 

## 5. Patents

This work led to Portuguese patent **PT118101** (2022/07/12). “**Hidrogéis Bioinspirados À Base De Polirotaxanos Deslizantes Funcionalizados Com Análogos De Catecóis, Processos De Produção E Suas Aplicações**”. **Inventors**: João Filipe Colardelle Da Luz Mano; Dora C.S. Costa; Maria Isabel Rial Hermida; João Manuel Marques Rodrigues. **Applicants**: University of Aveiro.

## Data Availability

The data presented in this study are available on request from the corresponding author. The data are not publicly available due to the patent in progress.

## Supplementary Materials

The following supporting information can be downloaded at: https://www.mdpi.com/article/10.3390/gels9020085/s1, Figures S1–S8: ^1^H and ^13^C NMR spectra, Figures S9 and S10: Rheological graphs, Video S1 (mp4) and Video S2 (mp4): Hydrogels.

## References

[1] Andersen A., Chen Y., Birkedal H. (2019). Bioinspired metal-polyphenol materials: Self-healing and beyond. Biomimetics.

[2] Azevedo S., Costa A.M.S., Andersen A., Choi I.S., Birkedal H., Mano J.F. (2017). Bioinspired Ultratough Hydrogel with Fast Recovery, Self-Healing, Injectability and Cytocompatibility. Adv. Mater..

[3] Andersen A., Krogsgaard M., Birkedal H. (2018). Mussel-Inspired Self-Healing Double-Cross-Linked Hydrogels by Controlled Combination of Metal Coordination and Covalent Cross-Linking. Biomacromolecules.

[4] Krogsgaard M., Nue V., Birkedal H. (2016). Mussel-Inspired Materials: Self-Healing through Coordination Chemistry. Chem. Eur. J..

[5] Lee H., Dellatore S.M., Miller W.M., Messersmith P.B. (2007). Mussel-Inspired Surface Chemistry for Multifunctional Coatings. Science.

[6] Mian S.A., Gao X., Nagase S., Jang J. (2011). Adsorption of catechol on a wet silica surface: Density functional theory study. Theor. Chem. Acc..

[7] Faure E., Falentin-Daudré C., Jérôme C., Lyskawa J., Fournier D., Woisel P., Detrembleur C. (2013). Catechols as versatile platforms in polymer chemistry. Prog. Polym. Sci..

[8] Yavvari P.S., Srivastava A. (2015). Robust, self-healing hydrogels synthesised from catechol rich polymers. J. Mater. Chem. B.

[9] Mamidi N., García R.G., Martínez J.D.H., Briones C.M., Martínez Ramos A.M., Tamez M.F.L., Del Valle B.G., Segura F.J.M. (2022). Recent Advances in Designing Fibrous Biomaterials for the Domain of Biomedical, Clinical, and Environmental Applications. ACS Biomater. Sci. Eng..

[10] Mamidi N., Delgadillo R.M.V. (2021). Design, fabrication and drug release potential of dual stimuli-responsive composite hydrogel nanoparticle interfaces. Colloids Surf. B Biointerfaces.

[11] Rial-Hermida M.I., Rey-Rico A., Blanco-Fernandez B., Carballo-Pedrares N., Byrne E.M., Mano J.F. (2021). Recent Progress on Polysaccharide-Based Hydrogels for Controlled Delivery of Therapeutic Biomolecules. ACS Biomater. Sci. Eng..

[12] Wang H., Heilshorn S.C. (2015). Adaptable Hydrogel Networks with Reversible Linkages for Tissue Engineering. Adv. Mater..

[13] Ding X., Wang Y. (2017). Weak bond-based injectable and stimuli responsive hydrogels for biomedical applications. J. Mater. Chem. B.

[14] Guyot C., Cerruti M., Lerouge S. (2021). Injectable, strong and bioadhesive catechol-chitosan hydrogels physically crosslinked using sodium bicarbonate. Mater. Sci. Eng. C.

[15] Holten-Andersen N., Jaishankar A., Harrington M.J., Fullenkamp D.E., Dimarco G., He L., McKinley G.H., Messersmith P.B., Lee K.Y.C. (2014). Metal-coordination: Using one of nature’s tricks to control soft material mechanics. J. Mater. Chem. B.

[16] Agergaard A.H., Pedersen S.U., Birkedal H., Daasbjerg K. (2020). Stimuli-responsive degrafting of polymer brushes: Via addressable catecholato-metal attachments. Polym. Chem..

[17] Shafiq Z., Cui J., Pastor-Pérez L., San Miguel V., Gropeanu R.A., Serrano C., del Campo A. (2012). Bioinspired underwater bonding and debonding on demand. Angew. Chem.-Int. Ed..

[18] Li J., Ejima H., Yoshie N. (2016). Seawater-Assisted Self-Healing of Catechol Polymers via Hydrogen Bonding and Coordination Interactions. ACS Appl. Mater. Interfaces.

[19] Costa A.M.S., Mano J.F. (2015). Highly robust hydrogels via a fast, simple and cytocompatible dual crosslinking-based process. Chem. Commun..

[20] Zheng S.Y., Liu C., Jiang L., Lin J., Qian J., Mayumi K., Wu Z.L., Ito K., Zheng Q. (2019). Slide-Ring Cross-Links Mediated Tough Metallosupramolecular Hydrogels with Superior Self-Recoverability. Macromolecules.

[21] Neto A.I., Vasconcelos N.L., Oliveira S.M., Ruiz-Molina D., Mano J.F. (2016). High-Throughput Topographic, Mechanical, and Biological Screening of Multilayer Films Containing Mussel-Inspired Biopolymers. Adv. Funct. Mater..

[22] Nakahata M., Mori S., Takashima Y., Yamaguchi H., Harada A. (2016). Self-Healing Materials Formed by Cross-Linked Polyrotaxanes with Reversible Bonds. Chem.

[23] Hart L.F., Tranquilli M.M., Rowan S.J. (2021). Material properties and applications of mechanically interlocked polymers. Nat. Rev. Mater..

[24] Hu W., Wang Z., Xiao Y., Zhang S., Wang J. (2019). Advances in crosslinking strategies of biomedical hydrogels. Biomater. Sci..

[25] Kobayashi Y. (2021). Precise synthesis of polyrotaxane and preparation of supramolecular materials based on its mobility. Polym. J..

[26] Kobayashi Y., Zheng Y., Takashima Y., Yamaguchi H., Harada A. (2018). Physical and adhesion properties of supramolecular hydrogels cross-linked by movable cross-linking molecule and host-guest interactions. Chem. Lett..

[27] Yokoyama N., Seo J.H., Tamura A., Sasaki Y., Yui N. (2014). Tailoring the supramolecular structure of aminated polyrotaxanes toward enhanced cellular internalization. Macromol. Biosci..

[28] Menyo M.S., Hawker C.J., Waite J.H. (2013). Versatile tuning of supramolecular hydrogels through metal complexation of oxidation-resistant catechol-inspired ligands. Soft Matter.

[29] Gomes M.C., Costa D.C.S., Oliveira C.S., Mano J.F. (2021). Design of Protein-Based Liquefied Cell-Laden Capsules with Bioinspired Adhesion for Tissue Engineering. Adv. Healthc. Mater..

[30] Mawani Y., Cawthray J.F., Chang S., Sachs-Barrable K., Weekes D.M., Wasan K.M., Orvig C. (2013). In vitro studies of lanthanide complexes for the treatment of osteoporosis. Dalt. Trans..

[31] Mayumi K., Liu C., Yasuda Y., Ito K. (2021). Softness, elasticity, and toughness of polymer networks with slide-ring cross-links. Gels.

[32] Mayumi K., Ito K. (2010). Structure and dynamics of polyrotaxane and slide-ring materials. Polymer.

[33] Maier G.P., Bernt C.M., Butler A. (2018). Catechol oxidation: Considerations in the design of wet adhesive materials. Biomater. Sci..

[34] Amaral A.J.R., Gaspar V.M., Mano J.F. (2020). Responsive laminarin-boronic acid self-healing hydrogels for biomedical applications. Polym. J..

[35] Minato K., Mayumi K., Maeda R., Kato K., Yokoyama H., Ito K. (2017). Mechanical properties of supramolecular elastomers prepared from polymer-grafted polyrotaxane. Polymer.

[36] Barrett D.G., Fullenkamp D.E., He L., Holten-andersen N., Lee K.Y.C., Messersmith P.B. (2013). pH-Based Regulation of Hydrogel Mechanical Properties through Mussel-Inspired Chemistry and Processing. Adv. Funct. Mater..

