# Supplementary material for: Bioinspired Oxidation-Resistant Catechol-like Sliding Ring Polyrotaxane Hydrogels
Source: Gels. 2023 Jan 19;9(2):85. doi: 10.3390/gels9020085 (PMC9956578; doi:10.3390/gels9020085)
Supplement: Supplementary file 1 [file gels-09-00085-s001.zip › gels-2164491-supplementary-Figures.pdf]

Supplementary Materials

Bioinspired Oxidation-Resistant Catechol-Like Sliding Ring Polyrotaxane Hydrogels

CONTENTS

1. SPECTRAL DATA 2

1.1 ^{1}H and ^{13}C NMR spectra of compounds 4a and 4b 2

1.2 Comparative ^{1}H NMR spectra of all derivatives 4

1.3 Rheological characterization 6

1. SPECTRAL DATA

1.1 ^{1}H and ^{13}C NMR spectra of compounds 4a and 4b

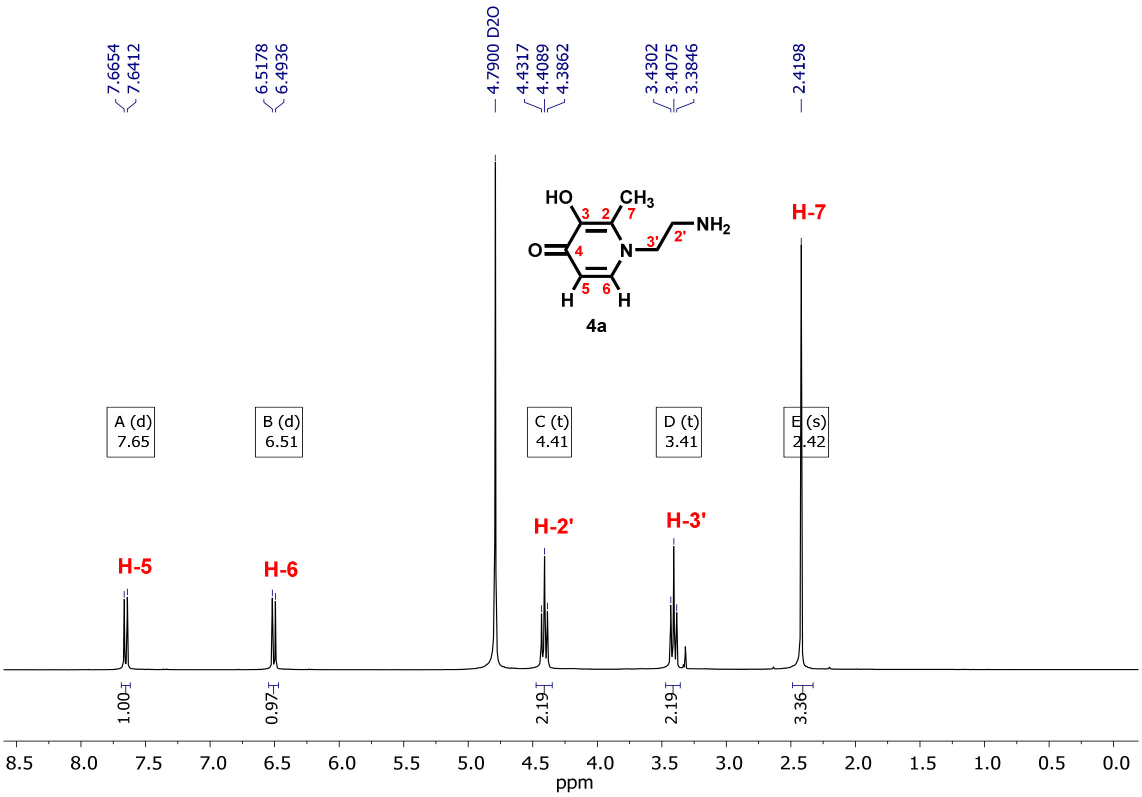

Figure S1. ^{1}H NMR Spectrum (300.0 MHz, D_2O) of HOPO-NH_2 (**4a**).

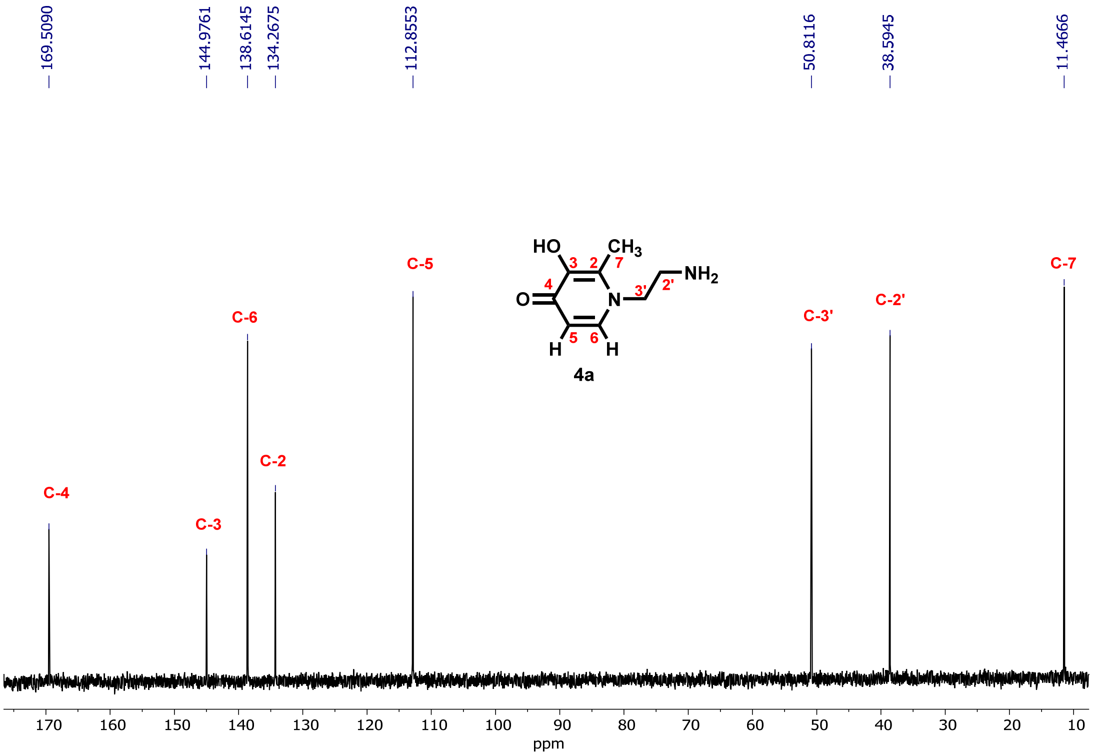

Figure. S2. ^{13}C NMR Spectrum (75.0 MHz, D_2O) of HOPO-NH_2 (**4a**).

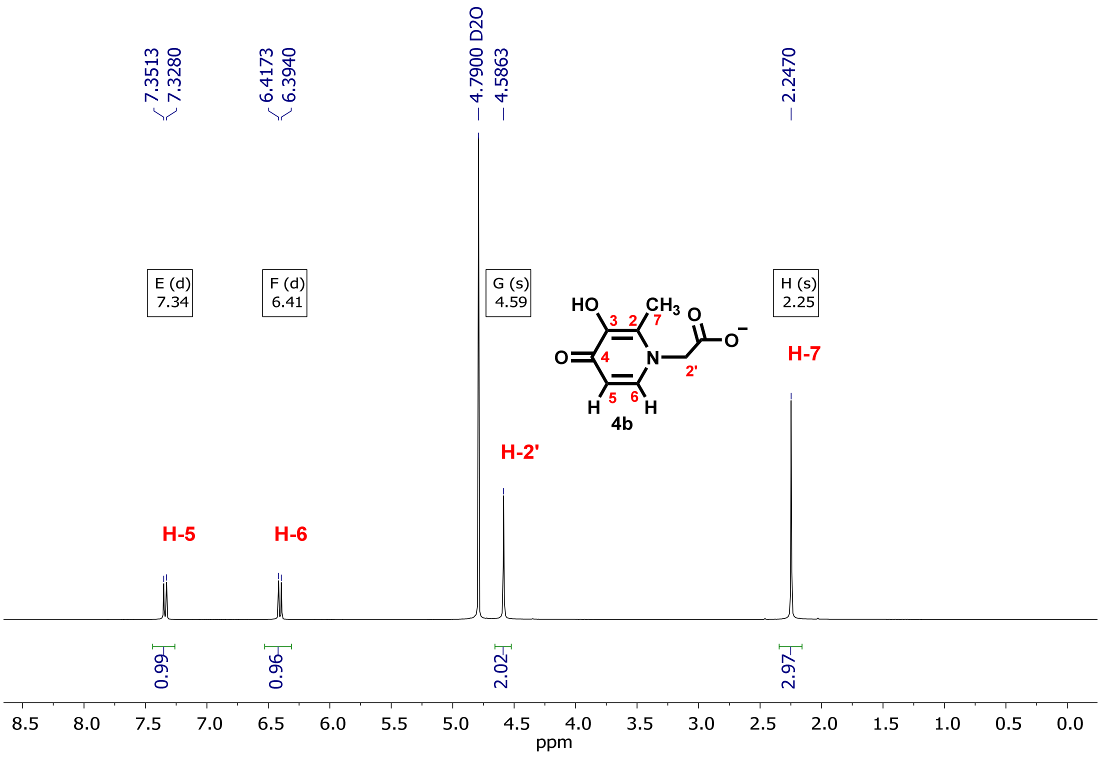

Figure S3. ^{1}H NMR Spectrum (300.0 MHz, D_2O+NaOD) of HOPO-CO_2H (**4b**).

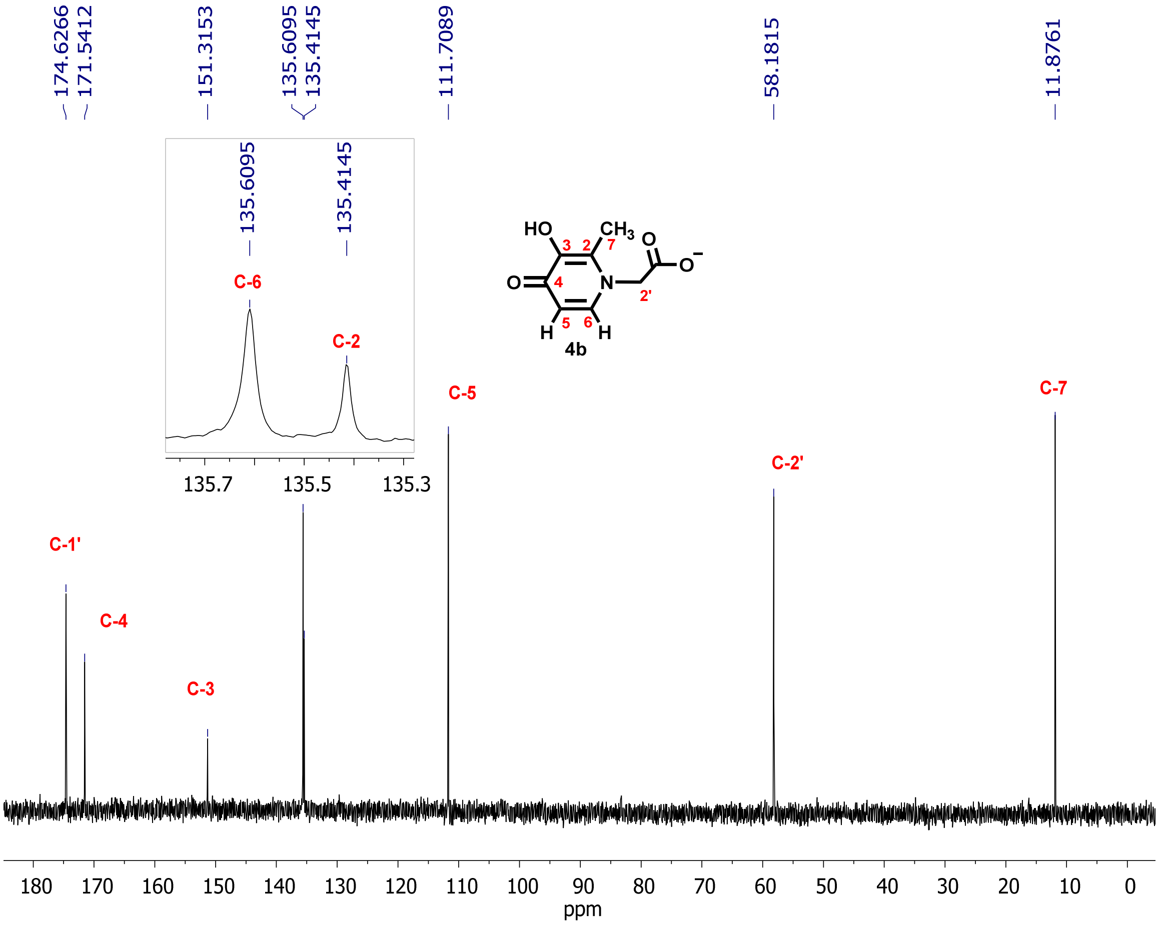

Figure S4. ^{13}C NMR Spectrum (75.0 MHz, D_2O+NaOD) of HOPO-CO_2H (**4b**).

1.2 Comparative ¹H NMR spectra of all derivatives

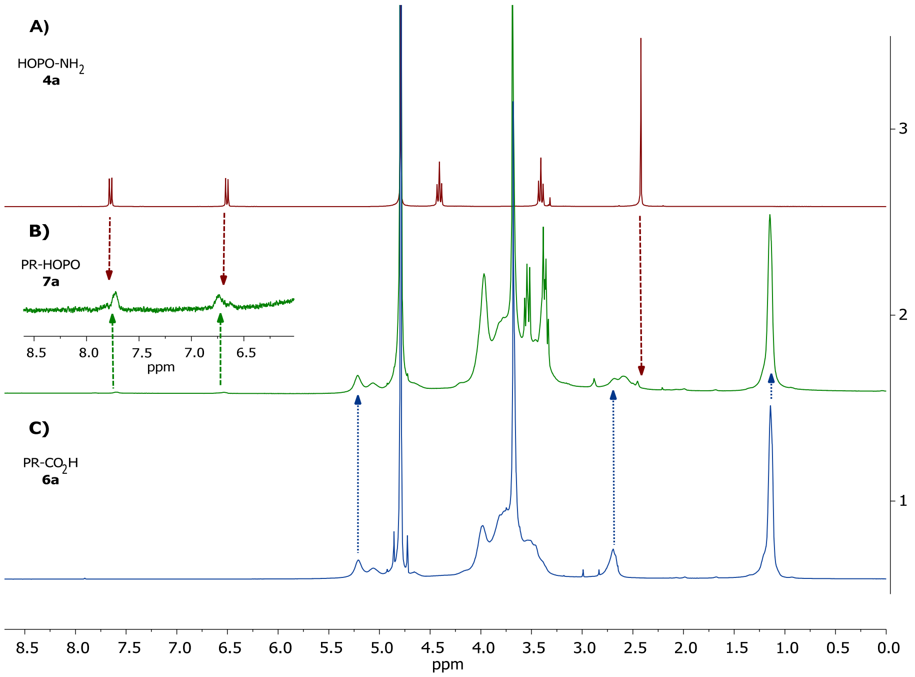

Figure. S5. Comparative ¹H NMR Spectra (D_2O, 300.0 MHz) of **A.** HOPO-NH_2 (**4a**), **B.** PR-HOPO **(7a)**, and **C.** PR-CO_2H (**6a**).

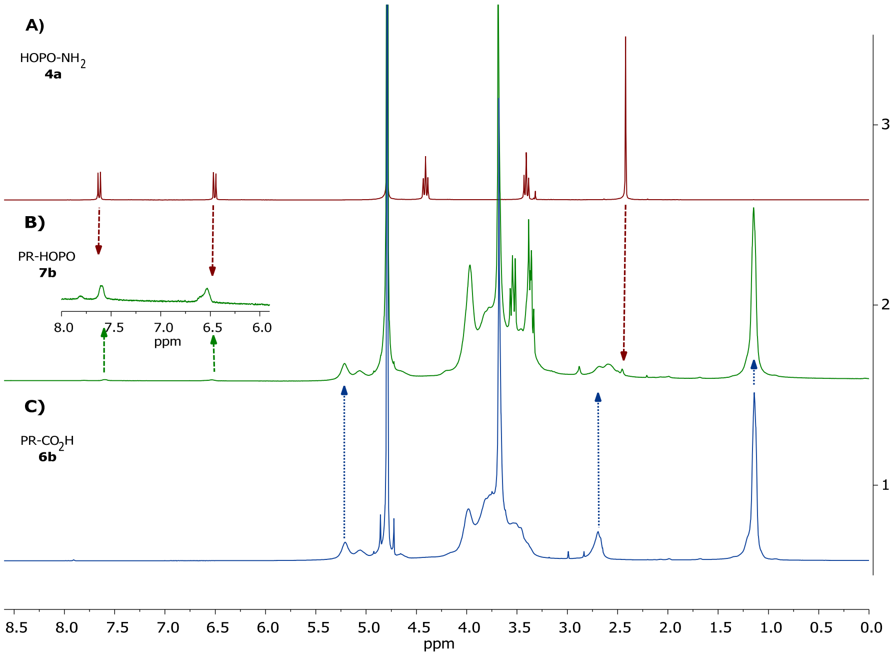

Figure. S6. Comparative ¹H NMR Spectra (300.0 MHz, D_2O) of **A.** HOPO-NH_2 (**4a**) **B.** PR-HOPO **(7b)** (**4a**) and **C.** PR-CO_2H (**6b**).

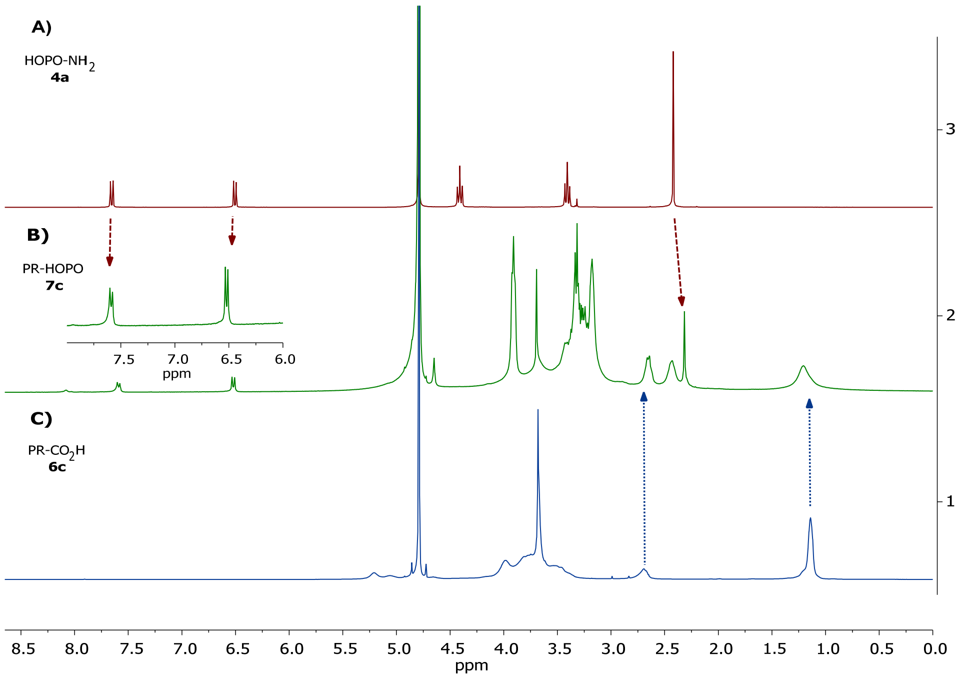

Figure. S7. Comparative ^{1}H NMR Spectra (300.0 MHz, D_2O) of **A.** HOPO-NH_2 (**4a**), **B.** PR-HOPO **(7c)** and **C.** PR-CO_2H (**6c**).

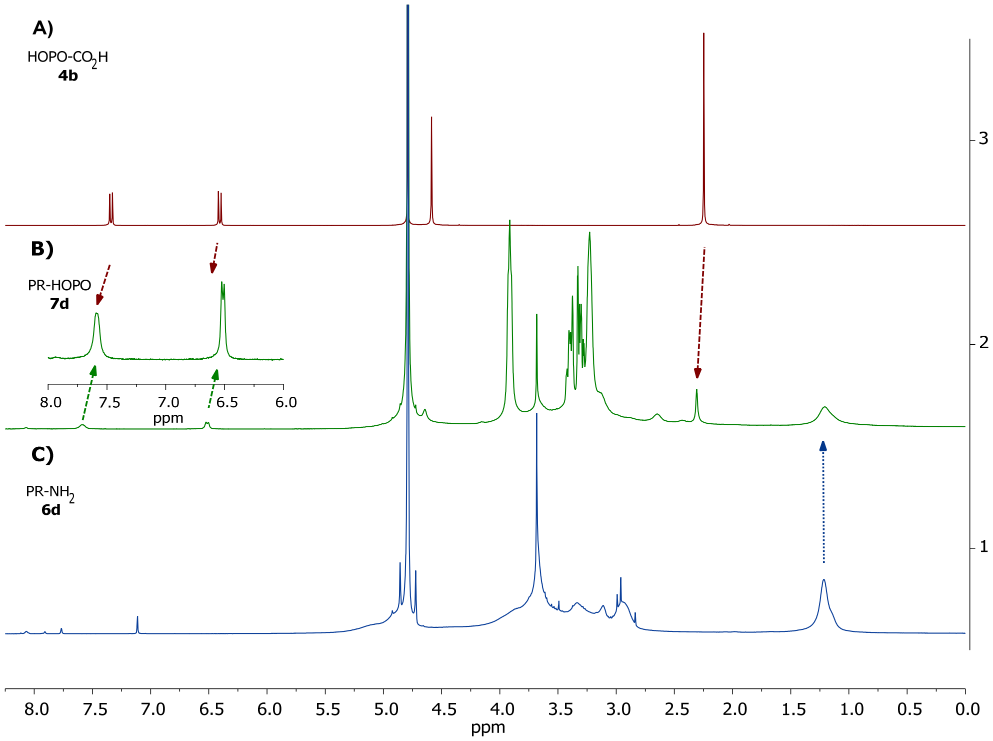

Figure. S8. Comparative ^{1}H NMR Spectra (300.0 MHz, D_2O) of **A.** HOPO-CO_2H (**4b**), **B.** PR-HOPO (**7d**) and **C.** PR-NH_2 (**6d**).

1.3 Rheological characterization

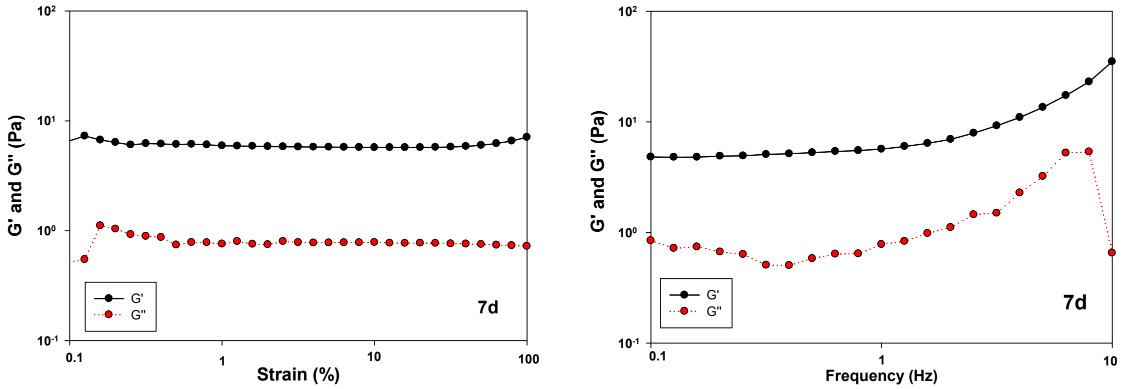

Figure. S9. Rheological properties of formulation based on $PRNH_2$ (**7d**). Strain sweep measurements at 1 Hz (fixed), and frequency sweep measurements at 1% of strain (fixed).

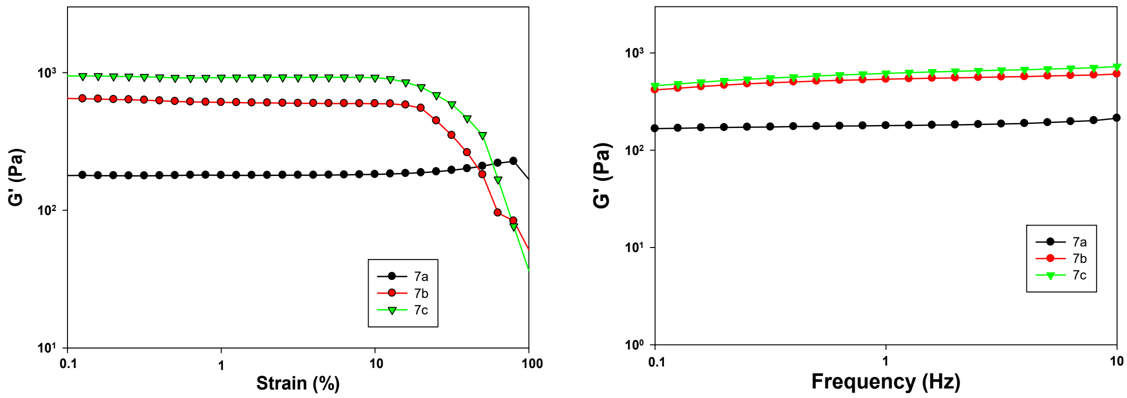

Figure. S10. Comparison of G′ moduli of the developed formulations (**7a–c**) at fixed frequency (1 Hz, left) and fixed strain (1%, right).
